# Comparison of the McGrath^® ^Series 5 and GlideScope^® ^Ranger with the Macintosh laryngoscope by paramedics

**DOI:** 10.1186/1757-7241-19-4

**Published:** 2011-01-17

**Authors:** Tim Piepho, Kathrin Weinert, Florian M Heid, Christian Werner, Rüdiger R Noppens

**Affiliations:** 1 Department of Anaesthesiology, University Medical Center of the Johannes Gutenberg-University-Mainz Langenbeckstr. 1, Mainz, 55131, Mainz, Germany

## Abstract

**Background:**

Out-of-hospital endotracheal intubation performed by paramedics using the Macintosh blade for direct laryngoscopy is associated with a high incidence of complications. The novel technique of video laryngoscopy has been shown to improve glottic view and intubation success in the operating room. The aim of this study was to compare glottic view, time of intubation and success rate of the McGrath^® ^Series 5 and GlideScope^® ^Ranger video laryngoscopes with the Macintosh laryngoscope by paramedics.

**Methods:**

Thirty paramedics performed six intubations in a randomised order with all three laryngoscopes in an airway simulator with a normal airway. Subsequently, every participant performed one intubation attempt with each device in the same manikin with simulated cervical spine rigidity using a cervical collar. Glottic view, time until visualisation of the glottis and time until first ventilation were evaluated.

**Results:**

Time until first ventilation was equivalent after three intubations in the first scenario. In the scenario with decreased cervical motion, the time until first ventilation was longer using the McGrath^® ^compared to the GlideScope^® ^and AMacintosh (p < 0.01). The success rate for endotracheal intubation was similar for all three devices. Glottic view was only improved using the McGrath^® ^device (p < 0.001) compared to using the Macintosh blade.

**Conclusions:**

The learning curve for video laryngoscopy in paramedics was steep in this study. However, these data do not support prehospital use of the McGrath^® ^and GlideScope^® ^devices by paramedics.

## Background

Endotracheal intubation remains the preferred technique to secure an airway during prehospital airway management [1]. Conventional direct laryngoscopy with a Macintosh blade is considered to be the standard technique for placing an endotracheal tube. Although adequate training in direct laryngoscopy is an important requirement for emergency medical personnel, the incidence of complications is still high, and the procedure is associated with a high mortality rate. During out-of-hospital emergencies, the incidence of unrecognised oesophageal intubation performed by paramedics has been reported to be as high as 16.7% [2,3].

In contrast to conventional direct laryngoscopy using a Macintosh blade, the novel technique of video laryngoscopy allows for a view of the glottis without requiring alignment of the oral, pharyngeal and laryngeal axes.

The McGrath^® ^series 5 video laryngoscope (Aircraft Medical Ltd, Edinburgh, UK) is a novel device designed for endotracheal intubation. It contains a small camera and a light source at the tip of the blade and therefore offers the user an image of the vocal cords and the surrounding airway anatomy on an LCD screen attached to the laryngoscope handle. The positioning of the McGrath^® ^blade tip is the same as a Macintosh blade. Once the glottis is visible on the monitor, an endotracheal tube is advanced through the vocal cords.

The GlideScope^® ^Ranger (Verathon Inc., Bothell, WA, USA) is a video laryngoscope with a separate monitor connected to the handle via a cable. The tip of the blade is equipped with a miniature camera and an LED light.

Several publications have shown a benefit with both instruments in expected and unexpectedly difficult airways when compared to a Macintosh laryngoscope in an in-hospital scenario [4,5]. Both devices are portable and could easily be included in emergency ambulance equipment inventories.

Until now, the efficacy and potential advantages of the McGrath^® ^Series 5 and the GlideScope^® ^Ranger in the hands of paramedics for the management of prehospital airways has not been fully evaluated.

With the objective of introducing alternatives to direct laryngoscopy during prehospital emergency medicine, we compared the two video laryngoscopes, the McGrath^® ^and the GlideScope^®^, with the Macintosh laryngoscope. Video laryngoscopy was evaluated during use by paramedics in an airway simulator. We hypothesised that the glottic view would be improved and that the airway would be more successfully secured using both video laryngoscopes compared to a Macintosh laryngoscope in a scenario with decreased cervical motion.

## Methods

Thirty board-certified paramedics participated in the study. Ethical approval was not considered necessary by the institutional review board. Prior to the study, each participant completed a questionnaire documenting his/her previous experience with the instruments. The paramedics were trained with the Macintosh laryngoscope, but none had any experience with the video laryngoscopes used in this study.

Each paramedic was given a hands-on standardised demonstration and verbal instructions for all devices by one of the investigators. A size 3 Macintosh blade, a size 3 GlideScope^® ^Ranger and the McGrath^® ^Series 5 video laryngoscope with an adjustable blade set in the middle position were used in this study. All endotracheal intubations were performed using a standard Magill 7.5-mm tracheal tube in a Laerdal ALS Simulator (Laerdal, Stavanger, Norway), which was positioned on the ground. For all intubation attempts, a malleable stylet was inserted in the endotracheal tube. When using both video laryngoscopes, the tube was bent into a "hockey-stick" curvature [6].

Each paramedic performed six intubations with all three laryngoscopes in a randomised order in a manikin with a normal airway. Balanced randomisation was derived using a random number generator http://www.graphpad.com. After the participants completed the sequence, they performed one endotracheal intubation with each device in a randomised order in the same manikin but with simulated cervical spine rigidity via a cervical collar (Ambu^® ^Perfit ACE; Ambu, Ballerup, Denmark).

The times required for successful endotracheal intubation in the normal airway and in the scenario with decreased cervical range were chosen as the primary endpoints. Moreover, the time until view of the glottis was achieved was also documented. This duration was defined as the time period from touching the handle of the laryngoscope until a comment by the participant that glottis view was achieved. The other time point documented was first ventilation. A common digital stop watch was used for all evaluations. A failed intubation was defined as an attempt in which endotracheal intubation was not successful or one that required > 120 s to perform. The quality of visualisation according to Cormack and Lehane [7] and the percentage of glottis opening (POGO) was evaluated [8].

After the participants completed the normal airway attempts, they were asked to score the degree of difficulty using each device on a scale from 1-6 (1 = excellent, 2 = good, 3 = satisfactory, 4 = sufficient, 5 = inadequate, 6 = fail). This procedure was then repeated after the decreased cervical motion scenario.

### Statistics

Data for POGO, time until glottic view and time until first ventilation were analysed using one-way analysis of variance (ANOVA) and a Bonferroni post-test. Exploratory comparisons between times to first ventilation within the groups were conducted with a two-way repeated-measure ANOVA and Bonferroni post-tests. Data from the success of tracheal intubation attempts were analysed using a Chi-square test; nonparametric data (Cormack & Lehane and rating) were analysed using the Kruskal-Wallis test and Dunn's post-test (GraphPad Prism version 5.00 for Mac, GraphPad Software, La Jolla, California, USA). Data are presented as mean ± SD or median (IQR [range]). A p-value of less than 0.05 was considered to be statistically significant.

## Results

Each of the paramedics completed the German paramedic course. Most of the participants performed a minimum of ten endotracheal intubations in patients.

### Normal airway

During the first intubation attempt, all participants successfully performed the endotracheal intubation with all three devices. Of all attempts, two intubation attempts failed while using the Macintosh laryngoscope. In addition, one intubation attempt using the McGrath^® ^video laryngoscope and one intubation attempt using the GlideScope^® ^were unsuccessful. There was no significant difference between the three laryngoscopes in regards to the success rate of tracheal intubation.

**Figure 1:**
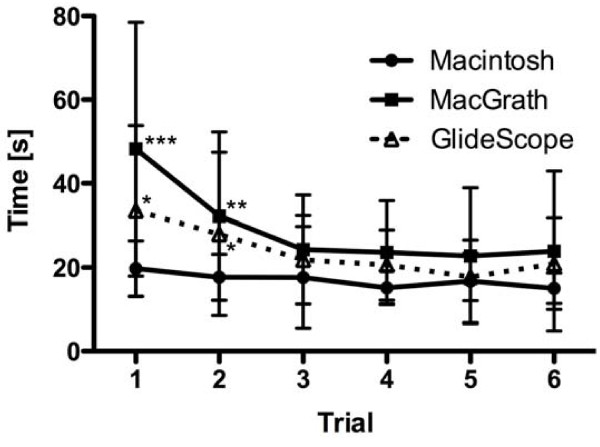
**Graphs representing the time until first ventilation for the Macintosh, the McGrath^® ^and the GlideScope^® ^laryngoscopes for all six attempts in a normal airway**. Mean ± SD. * = p < 0.05, ** = p < 0.01, *** = p < 0.001.

A learning curve was evident for both video laryngoscopes (Figure 1).

On the first trial, visualisation of the glottis was prolonged using the McGrath^® ^(12.6 s ± 9.1) compared to the GlideScope^® ^(9.6 s ± 6.6; p < 0.05) and Macintosh laryngoscopes (7.3 s ± 3.2; p < 0.01). On the second attempt, time to visualisation was similar among the devices (McGrath^® ^7.3 s ± 5.0; GlideScope^® ^6.7 s ± 3.5; Macintosh 6.8 s ± 3.8; p > 0.05). Following the first attempt, no differences were observed between the devices.

The total time to first ventilation during the first trial was faster using the Macintosh (19.8 s ± 6.6) compared to McGrath^® ^(48.2 s ± 31.1; p < 0.001) and GlideScope^® ^(28.6 s ± 14.0; p < 0.05; Figure 1). During the second attempt, the time to first ventilation with the Macintosh (17.7 s ± 5.5) was significantly faster compared to the McGrath^® ^(29.3 s ± 18.2; p < 0.01) and the GlideScope^® ^(25.7 s ± 18.3; p < 0.05) devices. No significant differences were observed on the following trials.

The quality of the laryngeal view varied among the three laryngoscopes (Tables 1 and 2). Both the McGrath^® ^and the GlideScope^® ^video laryngoscopes enabled a better glottic view than the Macintosh laryngoscope.

**Table 1:** Glottic view according to the Cormack & Lehane in a normal airway.

Trial	Macintosh	McGrath^®^	GlideScope^®^
1	2 (1-2 [1-2])	1 (1-2 [1-2])	1 (1-2 [1-3])
2	2 (1.75-2 [1-3])	1 (1-1.25 [1-3])	1 (1-2 [1-2])
3	2 (1-2 [1-2])	1 (1-1 [1-2])	1 (1-2 [1-2])
4	2 (1-2 [1-2])	1 (1-1 [1-2])	1 (1-2 [1-2])
5	2 (1-2 [1-3])	1 (1-1.25 [1-2])	1 (1-2 [1-2])
6	2 (2-2 [1-2])	1 (1-1.25 [1-2])	1 (1-2 [1-2])

Median (IQR [range]).

**Table 2:** Glottic view according to the POGO score (%) in a normal airway.

Trial	Macintosh	McGrath^®^	GlideScope^®^
1	72.8 ± 18	83 ± 21	81.5 ± 24.5
2	68.5 ± 22.9	87.2 ± 22.9	85.0 ± 19.7
3	76.7 ± 19.9	96.3 ± 6.1	83.5 ± 20.3
4	68.7 ± 23.6	90.5 ± 16.5	84 ± 18.2
5	62.7 ± 27.2	92.7 ± 10.8	79.5 ± 28.6
6	59.2 ± 24.5	92.7 ± 10.7	80 ± 25.2

Mean ± SD.

The paramedics rated the Macintosh a 2 (2-3 [1-4]), the McGrath^® ^a 2 (1-3 [1-5]) and the GlideScope^® ^a 2 (1-3 [1-5]), all similar, after the normal airway attempts.

### Scenario with decreased cervical motion

In the scenario with decreased cervical motion, all intubations were successful using the McGrath^® ^and the GlideScope^®^, and one attempt using the Macintosh laryngoscope failed.

The participants required 6.4 s ± 3.1 to adjust the view of the glottis with the Macintosh laryngoscope. No significant differences in the duration to glottic view were observed between the devices (McGrath^®^: 6.3 s ± 2.7; GlideScope^® ^7.3 s ± 5.4).

The time to first ventilation was prolonged using the McGrath^® ^(31.5 s ± 21.1) compared to the GlideScope^® ^(19.2 s ± 8.5; p < 0.01) and Macintosh (15.9 s ± 4.9; p < 0.001) devices.

Using the Cormack & Lehane classification, the McGrath^® ^offered a better view (rated 1 (1-2 [1,2])) than the Macintosh laryngoscope (2 (2-2 [1-3]); p < 0.001). However, no differences between the Macintosh and the GlideScope^® ^(2 (1-2 [1-3]) were noted.

In regards to the evaluation of glottic visualisation using the POGO score, glottic view was improved using the McGrath^® ^(85.2% + 14.7) and GlideScope^® ^(69.7% + 30.1) devices compared to the Macintosh (40.8% + 28.6; p < 0.001).

After the scenario with decreased cervical motion, the McGrath^® ^(1 (1-3 [1-3])) and the GlideScope^® ^(2 (1-3 [1-3)])) devices were rated superior in comparison to the Macintosh laryngoscope (2 (2-3 [2,3]); p < 0.01 and p < 0.05, respectively).

## Discussion

The learning curve for the use of both video laryngoscopes evaluated in this study among paramedics without any prior experience is steep. Glottic view was improved compared to Macintosh laryngoscopy, but no difference was noted in regards to the success rate.

Paramedics frequently perform emergency airway management as a potentially life-saving manoeuvre. Endotracheal intubation still remains the preferred route for securing an airway and providing ventilation in a prehospital setting. However, emergency tracheal intubation is frequently difficult to perform and is associated with a lower success rate compared to an in-hospital setting [9]. Repeated endotracheal intubation attempts increase airway-related complications such as hypoxia, pulmonary aspiration and adverse hemodynamic events [10]. Furthermore, failure of airway management may significantly increase morbidity and mortality [11,12]. These difficulties have led to the increased use of alternative supraglottic airway devices, such as the Combitube^® ^or Laryngeal Tube^® ^[13,14]. The most important advantages of these devices are their rapid learning curves [15]. However, all advantages of endotracheal intubation are not guaranteed, and supraglottic airways may fail as well. Only recently has it been proposed that in the absence of personnel skilled in endotracheal intubation, a supraglottic airway device is an acceptable alternative for ventilation in a pre-hospital setting [1].

Technical progress in regards to optical systems has facilitated the availability of different indirect laryngoscopes. The major advantage of these devices is that direct vision of the glottis is available. With the objective of pointing out alternatives to conventional direct laryngoscopy, we compared the McGrath^® ^and GlideScope^® ^video laryngoscopes with the Macintosh laryngoscope. However, we did not find significant differences in tracheal intubation success rates with the video laryngoscopes compared to the Macintosh blade in a normal airway or in a scenario with decreased cervical motion. This was due to the high tracheal intubation success rates with all devices in our study. One must consider the fact that the success rates for paramedical personnel performing endotracheal intubations using a Macintosh laryngoscope in similar studies are variable [16,17]. The reason for this variability may be attributed to different study settings. A wide variation in results has been described between different manikins when airway devices have been tested [18]. The repeated trials in the normal airway scenario demonstrated a reduced duration of intubation attempts for the GlideScope^® ^and McGrath^® ^devices compared to the first attempt. However, after the third trial, no further decrease in time to the first ventilation was observed. This confirms a rapid learning curve for the GlideScope^® ^and McGrath^® ^devices and is comparable to earlier studies [19-21]. In another publication, the learning curve for both video laryngoscopes in an airway simulator with a normal airway was studied. Sixty anaesthetists participated in the study. After five attempts, the time differences to successful endotracheal intubation persisted when compared with the Macintosh blade [21].

The time until glottic view in the second of the six "normal airway" attempts was nearly equal for all devices. However, the overall times to first ventilation for the McGrath^® ^and the GlideScope^® ^devices were longer when compared to the Macintosh laryngoscope. This was especially true for the McGrath^®^. Both video laryngoscopes enabled significantly better visualisation of the glottis. This is in accordance with previous studies. Although video laryngoscopes offer superior visualisation of the glottis, a good laryngeal view does not guarantee easy or successful tracheal tube insertion [22,23]. All video laryngoscopes without an integrated guide channel for the endotracheal tube could face the challenge of advancing the tube into the trachea. The tip of the tracheal tube must pass through an acute angle to enter the larynx and has a significant potential of coming in contact with the anterior tracheal wall [6].

Until the diagnosis of a cervical spine injury has been ruled out in a hospital, the cervical spine must be immobilised by a rigid collar. The limited mouth opening and limited neck extension results in a Cormack and Lehane grade 3 or 4 in 64% of these cases [24]. Therefore, this scenario is a typical difficult airway situation in a prehospital setting. The time to successful ventilation using the McGrath^® ^laryngoscope was significantly longer compared to the other laryngoscopes. Considering the time until glottic view, difficulty in passing the tube through the vocal cords was reconfirmed.

In their rating of the studied devices, the paramedics rated all three devices similarly after the normal airway trial. This might reflect familiarity with the Macintosh laryngoscope. However, when assessing their confidence in the use of each device for the scenario with decreased cervical motion, both video laryngoscopes were rated superior to the Macintosh laryngoscope. This result suggests that using the video laryngoscopes resulted in subjectively safer intubation.

Manikin studies have been proven to be a reliable surrogate for clinical scenarios. On one hand, a laboratory setting cannot simulate the precise conditions in an out-of-hospital patient; on the other hand, one advantage of manikin studies is that they allow for strict standardisation of study conditions. Therefore, the simulation of different intubation scenarios has been widely used in the past for similar studies [25-27]. Another limitation of this evaluation is the manikin used for this study. The ALS Simulator does not allow for the simulation of different difficult airway situations such as tongue oedema or limited jaw opening. The use of a cervical collar in this study did not result in a difficult airway that challenged the evaluated paramedics. Therefore, we could not fully evaluate the performance of the different devices in simulated difficult airway scenarios. Furthermore, we defined a maximal permissible duration of tracheal intubation attempt at 120 seconds. Without knowing the learning curve of both video laryngoscopes in the hands of paramedics, we wanted to evaluate the duration of the attempts without restricting time limitations.

## Conclusions

We conclude that the success rates of the McGrath^® ^and the GlideScope^® ^laryngoscopes were similar in comparison to the Macintosh laryngoscope, but the time to first ventilation was longer. Both video laryngoscopes exhibited a steep learning curve despite a deliberately brief instructional period and may enable a better view of the glottis over a conventional Macintosh laryngoscope when used by paramedics. These data do not support the prehospital use of the McGrath^® ^Series 5 and GlideScope^® ^Ranger devices by paramedics.

## Competing interests

The Surgical Company GmbH, Kleve, Germany provided the McGrath^® ^Series 5 video laryngoscope and Verathon Medical, Rennerod, Germany the GlideScope^® ^Ranger used in this study. The authors alone are responsible for the content and writing of the paper.

## Authors' contributions

TP has made substantial contributions to conception, acquisition of data and drafting the paper. KW was involved in conception and acquisition of data. FH has made substantial contributions to analysis and interpretation of data. CW has made substantial contributions to conception and revised the manuscript critically for important intellectual content. RN has made substantial contributions to conception, acquisition of data and revised the manuscript. All authors read and approved the manuscript.
